# Assessment of Two Audio-Recording Methods for Remote Collection of Vocal Biomarkers Indicative of Tobacco Smoking Harm

**DOI:** 10.1007/s40857-022-00279-0

**Published:** 2022-09-26

**Authors:** Marewa Glover, Marie-France Duhamel

**Affiliations:** 1 Centre of Research Excellence: Indigenous Sovereignty and Smoking, PO Box 89186, Torbay, Auckland, 0742 New Zealand; 2 Independent Researcher, Auckland, New Zealand

**Keywords:** Voice recording, Acoustic analysis, Vocal biomarkers, Larynx, Smoking

## Abstract

This study aimed to determine if self-complete at-home recordings could produce audio samples of sufficient quality for use in voice analysis software, and if audio samples of similar or sufficient quality could be extracted from audio-recorded naturalistic phone interviews. Data were obtained from 31 adults aged 18 years and over who smoked. The /a/ sound segment was manually isolated, and analysis functions were used to produce the following values: fundamental frequency, jitter, shimmer, noise ratio, formant 3, and formant 4. The /a/ sound segment was then manually isolated from audio recordings of naturalistic interviews previously conducted by phone. These were analysed in the same way and compared for quality against Evistr-recorded audio samples from the same participants. A third audio sample consisted of an Evistr or phone-recorded sustained phonation of the /a/ sound. Means and standard deviations were calculated for the target vocal parameters. Statistical comparisons for quality of sound segment were conducted for readings, interviews, and vowel phonation and for sound signals extracted via both recording methods. Self-recording by adults who smoked provided audio samples of sufficient quality for analysis of vocal features that have been associated with a clinical outcome. The values obtained for sustained phonation audio samples displayed the least perturbation and noise for the vocal parameters surveyed. Sound signals recorded with smartphones appeared to be affected by electronic interference but have potential for use in diagnostic tools for measuring vocal parameters.

## Introduction

### Voice Contributes to General Health

Speaking fundamental frequency (SFF) decreases as people age. Speaking frequencies are also affected by particular jobs. For example, a history of working in broadcasting has been associated with varying degrees of voice deterioration. While there are still many questions about the relative impact of voice and comorbid conditions on general health, it is known that people who smoke tobacco are at a higher risk of voice deterioration. Smoking irritates the lining of the larynx, which can cause dehydration that affects the free vibration of the vocal folds [1]. Smoking has a significant effect on laryngeal structures, leading to numerous health issues that include changes in the laryngeal area or more serious issues such as chronic inflammatory changes, which can lead to cancer and death [2]. Smoking can cause sinusitis, gastroesophageal disease, and respiratory disease, which includes emphysema, bronchitis, and cancer [3]. The level of deterioration of the laryngeal and voice is partly determined by how long a person has smoked, the quantity of cigarettes smoked, and alcohol consumption. For example, the risk of developing laryngeal cancer is greater among people who smoke more than 35 cigarettes a day [3].

### Larynx Cancer

The larynx, which is part of the throat situated at the entrance of the windpipe (trachea), plays an important role in breathing and speech. Larynx cancer is one of the most common cancers of the head and neck. Larynx cancer accounts for 1% of all new cancer cases and deaths globally. Between 1990 and 2017, the global cases of larynx cancer increased from 132,740 to 210,610 [4]. In New Zealand (NZ), in 2018 larynx cancer accounted for 1 per 100,000 population of new cancer cases, a slight decline from 1.2 per 100,000 in 2013 [5]. While tobacco smoking alone does not cause all larynx cancers, it is estimated that smoking increases the likelihood of developing larynx cancer.

### Increased Need for Remote Delivery of Healthcare

The widespread use of lockdown strategies, in response to the COVID-19 pandemic, has challenged the dominant face-to-face delivery mode of most healthcare services and research, including clinical trials. Lockdown measures included shelter-in-place orders for non-essential workers, that is, that they stay home and limit excursions beyond their property.

Remote health delivery presents a particular challenge for smoking cessation services and studies that use biofeedback tools, such as practitioner-administered exhaled carbon monoxide tests to motivate smoking cessation and validate smoking status and health improvement following abstinence. Some diagnostic sampling tools can be self-administered at home, such as cotinine in saliva tests, clipping a sample of hair, or collecting a urine sample. Postage and lab analysis costs can reduce accessibility to these methods. A further challenge is that most tests do not distinguish between nicotine use and tobacco smoking. Sending bodily samples to foreign laboratories for genetic analysis is an additional barrier to use of these methods. For example, some religious and cultural groups, including Indigenous peoples, have bioethical concerns about the use of their genetic information [6].

### Vocal Biomarkers

Voice represents a potentially low-cost, easily accessed, and non-invasive source for obtaining individual biomarkers to aid health diagnoses. The application of voice diagnosis technologies will have more uses and greater value globally if audio samples of sufficient quality can be collected remotely using a telephone or other digital platforms, and from naturalistic speech. Studies determining the acceptability, potential efficacy, and identifying parameters of relevance to different conditions in real-world settings are needed to advance this field.

This paper reports on a cross-sectional study conducted to inform the development and validation of a voice recording and analysis protocol for potential use in the future smoking cessation studies.

### Vocal Biomarkers of Smoking-Related Deterioration

The negative impact of smoking on voice has been demonstrated in two cross-sectional studies [7, 8] that compared vocal parameters in adults who smoked, and adults who did not smoke. Both studies analysed vowel sounds—[a] in Pinto et al. [7], and [æ], [i], and [ʊ] in Zealouk et al. [8]—in the speech of all participants. Both studies measured mean values for pitch (i.e. fundamental frequency F0), jitter, and shimmer, for the two groups of adults. Additionally, Zealouk et al. included formants values in their comparison, while Pinto et al. considered noise-to-harmonic ratio (NHR). Both studies observed that the values for fundamental frequency were lower for adults who smoke, whereas shimmer and jitter values are higher for the group of participants who smoke. It was also found that the presence of smoking can lower F3 and F4 values of formants and increase NHR values.

Another cross-sectional study on adults who smoked versus adults who had never smoked examined the early effects of smoking on the voice quality of 134 young women and men, aged 20–29 years (mean age = 22 years) [9]. Vocal parameters were measured for the fundamental frequency, jitter, shimmer, and noise-to-harmonic ratio for the /a/ sound. The values measured for the fundamental frequency F0, frequency perturbation parameters (jitter absolute ‘jitta’), and period-to-period perturbation quotients were noticeably affected by cigarette smoking, even among people who had smoked for 10 years or less. Fundamental frequency was significantly lower in young women who smoked, while frequency perturbation was significantly higher in young men who smoked. However, early stage of smoking was not found to have detrimental effect on the amplitude of sound signals (shimmer parameters) or to add noise-to-sound signals (NHR).

Therefore, we expect that we will similarly find that F0 and jitter parameters will be more sensitive to the effects of smoking than shimmer and NHR parameters, and that the effect of smoking on the F0 and jitter vocal parameters will differ by sex.

## Materials and Methods

### Design

This cross-sectional observation study aimed to determine if:

1. Self-complete-at-home recordings produce audio samples of sufficient quality for use in voice analysis software Praat [10] and
2. Audio samples of similar or sufficient quality for use in Praat can be extracted from audio-recorded naturalistic phone interviews.

A third objective was to identify optimum voice parameters to inform the design of research on the intervention potential of voice deterioration information for enhancing risk perception and motivation to stop smoking.

### Participants

Participants were 49 NZ adults aged 18 years and over who smoked and had no immediate intent to stop smoking. They were drawn from a larger four-year longitudinal qualitative study aimed at identifying barriers to stop smoking.

### Recruitment (into the Voices of the 5% Study)

Advertisements for Voices of the 5% Study participants were placed in NZ print and online media, including social media. Similarly, notices were distributed to the researchers’ networks. A snowball method was also used, whereby enrolled participants were asked to send the study advertisement or notice to people they knew who might be eligible.

Potentially eligible respondents were sent a participant information sheet (PIS) and consent form by email or post. The PIS explained the purpose of the study, what participants would be asked to do, who was conducting the research, and who was funding the study. The PIS advised the participants that they would be asked to record their voice on a portable handheld audio recorder. The PIS stated that: all information collected from participants would be treated in the strictest confidence; no individual would be able to be identified at any stage in the publication or presentation of the study’s findings; all participant information sheets would be kept in a locked cabinet separate from de-identified collected data; and that, following publication of the results of the study, the raw data would be made available online. In addition, participants were informed that they could withdraw from the study at any time without having to give a reason. The date of the withdrawal and any reasons given for withdrawal, if provided, were recorded in the study notes. Recruitment ran from July 2020 to February 2021.

If participants returned a signed informed consent form they were enrolled into the study and a baseline interview was conducted.

### Vocal Biomarker Materials

The audio recorder package provided to each participant consisted of an Evistr Voice Recorder (model L157), earphones, USB cable, user manual, instruction sheet, and script to be read.

### Script

Participants were asked to read the *Rainbow Passage*. This script was chosen for its sound repetition, unusual consonants and vowel combinations, and having short and long passages that help to test speech patterns [11].

### Data Collection Procedure

Demographic information (age, sex) and history of smoking data (age began regular smoking, total estimated time off smoking) were extracted from the Voices of the 5% Study baseline interview data.

Participants were informed that a recorder was going to be sent to them and they were given an overview of the instructions for its use. The recorder, task instructions, and return-addressed courier bag were then sent to each participant.

**Fig. 1:**
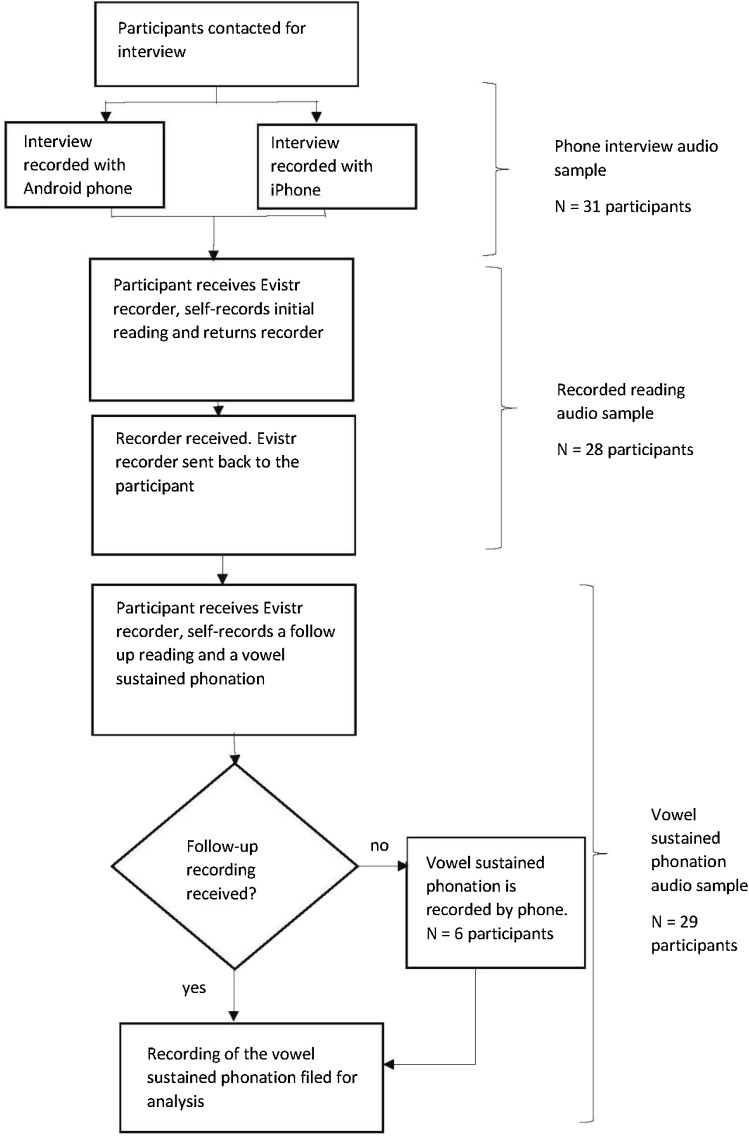
Data collection flowchart

Participants’ baseline interviews generally took place before their initial recording of reading the *Rainbow Passage*. Recordings of the vowel sustained phonation took place at a later date—for most participants at the end of a follow-up reading. The chronology of data collection is presented in Fig. 1.

#### Recorded Reading

The instruction sheet covered how to use the recorder, to ensure there was no background noise, to sit in a relaxed position with their chin up, and to say their first name, the time, and what room of their house they were sitting in. When prepared as instructed, the participants recorded themselves reading the *Rainbow Passage* at a moderate pace, twice.

Upon their return, the audio recordings were then downloaded and filed as.wav files, bit rate 1536 kbps.

#### Phone Interview

The second data source for this study was naturalistic phone interviews with Voices of the 5% Study participants. These were conducted by smartphones using a recorder application. Due to Apple deleting the previously inbuilt phone call recording app, interviews conducted via iPhone used the speaker function on the phone while holding an Evistr recorder close to the iPhone.

#### Vowel Sustained Phonation

In a recording exercise performed after the initial recording, specifically for this study, the recorders were returned to participants in the same manner. Participants were instructed to emit the /a/ sound in a sustained manner after reading the *Rainbow Passage* once. Vowel phonation was recorded by phone for six participants whose Evistr-recorded audio samples were not returned in time for analysis.

Phone-recorded audio samples from interviews and sustained vowel were all analysed as part of the phone-recorded data set.

### Vocal Parameters

The set of vocal parameters measured were fundamental frequency, jitter, shimmer, noise, and formants.

#### Fundamental Frequency (F0)

**Fig. 2:**
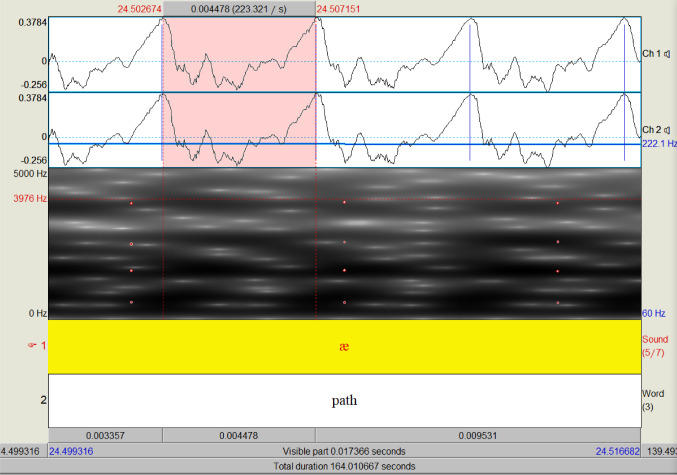
Screenshot of PRAAT software: sound waveform (top), spectrogram (bottom). For the highlighted cycle, Period Length *T* = 0.004478 s, *F*0 = 223.321

The fundamental frequency (F0) of a voiced sound is the frequency, or number of oscillations per second, at which the vocal cords vibrate when a human produces a sound (illustrated in Fig. 2). It is often referred to as pitch, which is our perception of the fundamental frequency, and F0 is expressed in Hertz (Hz). Fundamental frequency ranges between 80 and 450 Hz, is typically lower for males than females, and fluctuates within speech for the same individual.

Cigarette smoking has been observed to have an impact on fundamental frequency. Specifically, the value of this acoustic parameter decreases [7, 8, 12] as a result of the effect of smoking on vocal cords. Gonzalez and Carpi [9] observed that this parameter was especially affected by smoking in women and among people who had smoked for 10 years or less.

We anticipated observing low fundamental frequency in participants with a long history of smoking.

#### Jitter

**Fig. 3:**
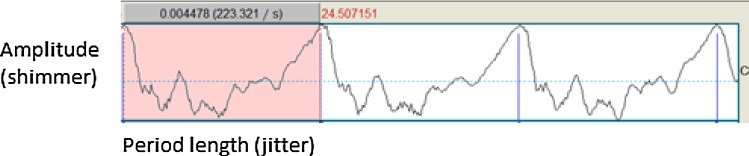
Sound waveform: three cycles of fundamental frequency, with amplitude (shimmer) and period length (jitter) highlighted for one cycle

Jitter is the amount of variation in period length of the fundamental frequency (illustrated in Fig. 3). Jitter parameters assess perturbation in the frequency of the sound signal and indicate vocal deterioration caused by a lack of control of vocal fold vibration [13], an abnormality that has been observed during phonation in people who smoke [14]. Jitter values have been found to be higher in people who smoke [7, 8, 15].

We measured four different parameters of this variation: its absolute period-to-period difference (jitter absolute, jitta) and three mean period-to-period difference scores: relative period-to-period variability of the pitch period (jitt), period-to-period relative average perturbation (rap), and the pitch perturbation quotient within 5 periods (ppq5).

#### Shimmer

Shimmer is the amount of variation in amplitude of the fundamental frequency (illustrated in Fig. 3). Shimmer assesses perturbation in the amplitude of consecutive periods and indicates vocal deterioration caused by lesions in the vocal cords [13]. Amplitude abnormalities were observed during phonation in the voice of people who smoke [14], and shimmer values have been found to be higher in people who smoke [7, 8].

We measured four different parameters of this variation: its cycle-to-cycle difference in decibels (shdB) and three mean cycle-to-cycle difference scores: relative evaluation of cycle-to-cycle variability of amplitude (shim), amplitude perturbation quotient over three periods (apq3), and amplitude perturbation quotient over five periods (apq5).

#### Noise ratio

The noise-to-harmonic ratio (NHR) detects the presence of noise in the sound signal, and it is measured in decibels (dB). A higher NHR indicates worse voice quality. Current smoking is associated with an increase in the NHR [7].

No association has been documented between cigarette smoking and a change in the value of the harmonic-to-noise ratio (HNR), which reflects the efficiency of speech. According to Teixeira et al. [15], “a value of less than 7 dB in HNR is considered pathological”.

We were interested in HNR because it has been measured in a previous study assessing the effect of vaping on voice quality [16]. Tuhanioğlu et al. [16] found that mean HNR values among people in the e-cigarette group and the control group of people with no history of smoking were higher than the mean HNR value for people who smoked. It was worth monitoring HNR values in our study because some of our participants reported switching to e-cigarettes during the study.

#### Formants

**Fig. 4:**
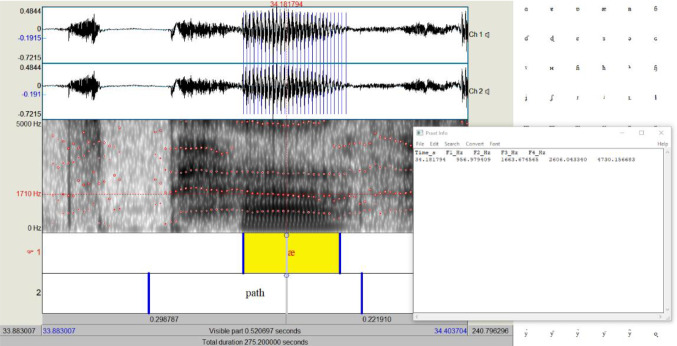
Red lines on the spectrograph of sound [æ] indicate the four formants for this sound in this recording, from Formant 4 (*F*4), top line, to Formant 1 (*F*1), bottom line. The formant values at cursor point (34.18), about midway in the highlighted sound signal, are given in the window on the right. We are interested in the values of *F*3 and *F*4, respectively, 2606.04 Hz and 4730.15 Hz, in this example

Formants indicate the resonant frequencies of the vocal tract. For vowels, this information is relayed in four formants: F1, F2, F3, and F4 (illustrated in Fig. 4). Zealouk et al. [8] observed little correlation between current smoking and values of F1 and F2. However, F3 and F4 values for the vowels they investigated (/a/, /i/, and /u/) were lower in people who smoke. Therefore, we monitored the values of the two last formants, F3 and F4.

## Data Analysis

### Sound Signal Investigated: /a/

Vowels are voiced sounds, and they are released with an acoustic energy that is not restricted by articulation, unlike what happens for many voiced consonants. Based on these characteristics, vowels are the ideal segments for assessing voice quality and measuring acoustic parameters relating to the vocal tract.^1^

We used Version 6.1.42 of the Praat software [10] to analyse the vocal parameters under investigation in this study: fundamental frequency (F0), jitter, shimmer, formants (F3 and F4), and noise ratio (NHR and HNR).

Studies by Zealouk et al. [8] and Pinto et al. [7] both selected the /a/ sound for their analysis of vocal parameters. Zealouk et al. extracted tokens of the vowel sound from recorded speech, while Pinto et al. analysed the sustained emission of the /a/ sound by their participants. Both studies provide us with a reference point for this specific sound. Additionally, similar to Zealouk et al., we extracted the /a/ sound from recorded naturalistic speech, and then, like Pinto et al., we analysed the sustained phonation of /a/ recorded by the participants.

### Segments Extraction from the *Rainbow Passage*

For each recording of participants reading the *Rainbow Passage*, we extracted tokens of the /a/ sound segments from the word ‘path’ contained in the following paragraph (emphasis added):

*When the sunlight strikes raindrops in the air, they act as a prism and form a rainbow. The rainbow is a division of white light into many beautiful colours. These take the shape of a long round arch, with its ****path*** *high above, and its two ends apparently beyond the horizon.*

In NZ English, /a/ in the word ‘path’ tends to be released as a long vowel, which allowed for taking measures in the central part of the vowel, thus avoiding influence of co-articulation from surrounding sound segments. Furthermore, ‘path’ is a common word, on which participants were unlikely to stumble. Also, the word occurs some distance into the recorded speech when the speaker’s voice and speech delivery had stabilised. The vowel in the word ‘path’ may be released as [a:], [a], or [æ], a variability that may present a limitation in any study that surveys sound segments extracted from natural speech. This variability, expected to be participant-specific, and therefore carried over time in subsequent recordings, would, however, be reflected in formants F1 and F2, which were not used in our measures, since they had not been revealed to be affected meaningfully by cigarette smoking [8].

### Segments Extracted from Interviews

For each of the participants who provided a usable Evistr recording, a matching /a/ sound segment in the word ‘car’, or ‘start’, was isolated in their audio-recorded Voices of the 5% baseline interview. The same vocal parameters were analysed, and then, the Evistr and Voices interview samples were compared.

### Analysis of Sustained /a/ Sound

Participants were asked to record themselves emitting a sustained /a/ sound using the Evistr recording device; for six participants, the sustained sound was phone-recorded.

### Parameters Analysis

#### Fundamental Frequency F0

Fundamental frequency analysis was controlled for sex/gender for three reasons. Firstly, because fundamental frequency was expected to be higher for women than men. Secondly, because this frequency is especially affected in women, even among people who had smoked for 10 years or less [9]. Thirdly, two of our participants were non-binary and at different stages in following a hormonal treatment that is likely to affect their vocal fundamental frequency.

#### Jitter and Shimmer

Jitter parameters assess perturbation in the frequency of a sound signal, while shimmer values assess perturbation in its amplitude. Smoking increases jitter and shimmer values.

#### Noise

Smoking is associated with an increase in the noise-to-harmonics ratio (NHR), which detects the presence of noise in the sound signal. The harmonic-to-noise ratio (HNR) reflects the efficiency of speech and increases with vaping.

#### Formants F3 and F4

Similar to the fundamental frequency, the values of F3 and F4 formant frequencies were expected to be lower for men than women, and the presence of smoking has been observed to decrease their values.

### Comparison of the Recording Type

The R function *aov* was used to compute the variance between the means for the three types of audio samples, and between the means for the different types of recording gear. Pairwise comparison (function *TukeyHD)* was used to determine whether means between the three pairs of audio sample and means between phone-recorded and Evistr-recorded signals were statistically significant. In this analysis, the p value measures the probability that the difference in means could have occurred by chance, the lower the p value, the greater the statistical significance of the mean difference.

## Results

### Response Rate

Of a total of 49 participants who were sent a recorder, 31 participants returned their initial recordings. Two participants withdrew from the study and 16 did not return their recorder by courier or it arrived too late to be included in this analysis.

### Participant Characteristics

Participant demographics are summarised in Table 1.

**Table 1:** Participant demographics at baseline

Participants	Number	Age range	Mean age	Number, age ≤ 40	Number, age > 40	Mean years smoking
Women	16	24–63	36	12	4	19.5 (5–45)
Men	13	19–81	48	6	7	31.5 (2–63.5)
Non-binary	2	26–30	28	2	0	13 (10.5–15)
All	31	19–81	40.5	20	11	24 (2–63.5)

### Recording Results

All but one participant recorded two readings of the *Rainbow Passage*. The final 61 readings, all captured on Evistr recorders, provided sufficient material to calculate the mean and standard deviation values of the target vocal parameters from the extracted sound signal.

The phone interviews of three participants provided no usable tokens of the /a/ sound segment in the words ‘car’ or ‘start’. This happened when there was loss of sound signal during the phone interview, or because the participant’s answer was very concise. Two participants provided no recordings of the vowel sustained phonation, and for six participants, the recording was made via smartphone, rather than with the Evistr audio recorder. Table 2 displays the mean and standard deviation (SD) values of the vocal parameters for the /a/ sound for the three types of audio samples.

**Table 2:** Vocal parameters for sound signal /a/ analysed in baseline readings, interviews, and sustained phonation

Vocal parameters	*F*0	jitt (%)	jitta (µs)	rap (%)	ppq5 (%)	shim (%)	shdB (dB)	apq3 (%)	apq5 (%)	NHR	HNR	*F*3	*F*4
Recorded readings—participants *n* = 31, analysed tokens *n* = 61, recorded: EVISTR audio recorder, date range Nov 2020–March 2021													
Mean	160	1.10	78.70	0.49	0.54	6.11	0.57	2.71	3.37	0.12	12.85	2653	3660
SD	42.5	0.84	68.00	0.45	0.41	3.12	0.28	1.56	1.85	0.09	3.88	248	244
Interviews—participants *n* = 28, analysed tokens *n* = 66, recorded: smartphone + recording application, date range July 2020–April 2021													
Mean	151	1.49	110.85	0.58	0.64	8.58	0.76	4.36	*	0.23	10.00	1974	2826
SD	43.3	1.11	84.07	0.65	0.46	2.99	0.26	2.12	*	0.20	3.75	364	407
Sustained phonation—participants *n* = 29, analysed tokens *n* = 29, recorded: EVISTR audio recorder**, date range June 2021–August 2021													
Mean	170	0.54	34.60	0.27	0.30	4.07	0.37	2.12	2.47	0.05	17.42	2599	3689
SD	43.7	0.44	28.10	0.23	0.23	2.99	0.27	1.55	1.81	0.05	4.58	494	539

*Shimmer apq5% values were indeterminate for the phone-recorded audio samples of several participants’ interview

**For 6 participants, sustained /a/ was recorded with a smartphone using a recording application

Table 3 displays the difference in means and statistical significance (p value), by pair of audio samples (Table 3).

**Table 3:** Vocal parameters: Comparison of means and statistical significance, by pair of audio samples

Vocal parameters	*F*0	jitt (%)	jitta (µs)	rap (%)	ppq5 (%)	shim (%)	shdB (dB)	apq3 (%)	apq5 (%)	NHR	HNR	*F*3	*F*4
*Recorded readings—interviews*													
Means difference	8.80	− 0.38	− 32.10	− 0.08	− 0.10	− 2.46	− 0.19	− 1.64	*	− 0.10	2.84	679	834
*p* Value	= 0.71	= 0.18	= 0.14	= 0.75	= 0.52	< 0.01	< 0.05	< 0.05	*	< 0.01	< 0.05	< 0.001	< 0.001
*Recorded readings—sustained phonation*													
Means difference	− 9.53	0.55	44.19	0.21	0.23	2.04	0.18	0.58	0.89	0.07	− 4.58	54	− 29
*p* Value	= 0.66	< 0.05	< 0.05	= 0.18	< 0.05	< 0.05	< 0.05	= 0.4	= 0.32	= 0.08	< 0.01	= 0.84	= 0.95
*Interviews—sustained phonation*													
Means difference	− 18.3	0.94	76.29	0.30	0.34	4.50	0.38	2.23	*	0.18	− 7.42	− 624	− 863
*p* Value	= 0.24	< 0.001	< 0.001	< 0.05	< 0.01	< 0.001	< 0.001	< 0.001	*	< 0.001	< 0.001	< 0.001	< 0.001

*Shimmer apq5% values were indeterminate for the audio samples of several participants’ interview

Table 4 displays the mean values of the vocal parameters for the audio signal recorded by smartphone or audio recorder, the difference in means between the two types of recording equipment, and the statistical significance of this difference.

**Table 4:** Measures of vocal parameters analysed by recording equipment

Vocal parameters	*F*0	jitt (%)	jitta (µs)	rap (%)	ppq5 (%)	shim (%)	shdB (dB)	apq3 (%)	apq5 (%)	NHR	HNR	*F*3	*F*4
*Audio recorder: n* = *54*													
Mean	164	0.84	58.8	0.38	0.42	5.00	0.46	2.34	2.84	0.08	14.8	2703	3762
SD	43	0.72	58.4	0.37	0.34	3.21	0.29	1.56	1.86	0.08	4.72	303	321
*Phone recorded: n* = *34*													
Mean	155	1.36	99.2	0.55	0.60	8.16	0.72	4.15	*	0.20	11.2	1968	2835
SD	44	1.09	81.8	0.61	0.46	3.11	0.27	2.07	*	0.19	4.83	361	380
*Means difference*													
(phone means—recorder means)	− 9	0.52	40.4	0.17	0.18	3.16	0.26	1.81	*	0.12	− 3.6	− 735	− 927
*p* Value	= 0.374	< 0.01	< 0.01	= 0.124	< 0.05	< 0.001	< 0.001	< 0.001	*	< 0.001	< 0.001	< 0.001	< 0.001

*Shimmer apq5% values were indeterminate for the audio samples of several participants’ phone recorded interview

Furthermore, audio samples provided for a comparison of the means of the vocal parameters measured for vowel phonation recorded via smartphone (six participants) and Evistr recorder (23 participants). The differences in means showed that phone-recorded audio samples displayed (a) highly significant lower values for formants F3 (difference =  − 829; *p* value < 0.001) and F4 (difference =  − 1020; *p* value < 0.001) and (b) significant higher shim% value (difference =  + 2.73; *p* value < 0.05) and shim apq5 value (difference =  + 1.60; *p* value < 0.05). Means for the other vocal parameters showed no significant difference.

#### Fundamental and Formants Frequencies

The three tables below display mean and standard deviation values for the fundamental and formant frequencies by sex/gender, for recorded readings (Table 5), phone interviews (Table 6), and sustained phonation (Table 7).

**Table 5:** Recorded readings: vocal frequency values by gender

Vocal frequencies	*F*0	*F*3	*F*4
*Women*			
Mean	189.18	2764.15	3661.47
SD	28.72	270.96	337.53
*Men*			
Mean	123.34	2512.44	3618.18
SD	27.93	214.94	242.54
*Non-binary*			
Mean	157.08	2621.46	3921.79
SD	51.07	586.35	828.39

**Table 6:** Phone interviews: vocal frequency values by gender

Vocal frequencies	*F*0	*F*3	*F*4
*Women*			
Mean	170.55	1854.75	2630.99
SD	35.59	341.64	252.04
*Men*			
Mean	132.91	2009.95	2868.70
SD	41.96	337.37	321.54
*Non-binary*			
Mean	145.56	2514.82	3809.05
SD	70.96	126.92	168.59

**Table 7:** Sustained phonation: vocal frequency values by gender

Vocal frequencies	*F*0	*F*3	*F*4
*Women*			
Mean	195.01	2681.50	3830.91
SD	28.14	533.52	444.88
*Men*			
Mean	134.28	2548.46	3540.83
SD	41.24	367.70	651.58
*Non-binary*			
Mean	160.96	2213.92	3374.51
SD	20.41	882.76	386.75

## Discussion

The aim of this study was to determine if (a) audio samples of sufficient quality could be collected at a distance for analysis in Praat software and (b) these audio samples could be collected from naturalistic speech via smartphone or other digital platforms. The study also aims to identify the optimum voice parameters to assess voice quality.

### Comparing Audio Samples

Reported values for the vocal parameters we surveyed show a gradation for the three audio samples. The sustained phonation audio samples were of the highest quality, with values consistently tending toward those of healthy voices [13]. In contrast, phone interview samples return values that are the closest to those found in people who smoke [7–9]. In the middle, the measures obtained for recorded readings range between those of the two other audio samples.

This gradation is remarkably consistent across parameters, and the difference in means is highly significant between sustained phonation and interviews. Sustained phonation samples show (a) the highest frequency values (*F*0 and formants *F*3 and *F*4) and speech efficiency (harmonic-to-noise ratio), (b) the lowest frequency perturbation (jitter, shimmer), and (c) the presence of noise in the sound signal (NHR). This pattern is reversed for phone interview samples. As for recorded readings, values obtained for formants *F*3 and *F*4 show no significant difference with those obtained for sustained phonation, while *F*0 and jitter values show no significant difference with values observed for interviews. Shimmer and NHR values sit midway between values of the two other audio samples, and HNR is closer to the audio samples extracted from the interviews.

The acoustic characteristics of the sustained phonation of the /a/ sound gave overall measures that correlate with voices healthier than in the two other audio samples. This is probably due to the controlled emission of the participant’s voice recorded in this sample, which departs from the naturalistic speech segments extracted from the two other samples. These other samples are affected by the variability that typically occurs in naturalistic speech.

### Effect of Recording Equipment

The measures of vocal parameters analysed by recording equipment suggest that sound signals recorded by smartphones amplify the perturbations detected in acoustic characteristics of voice. In comparison with Evistr recordings, the overall measures for smartphone-recorded signals show (a) a pattern of significantly lower *F*3, *F*4, and HNR values and (b) significantly higher shimmer and NHR values. Used as a one-off, these measures may erroneously point to an unhealthy voice, but they could be used in a longitudinal study to track variability in acoustic characteristics over time.

#### Type of Phone Recording Device and Application

The sound quality of the participants’ voice was uneven across phone-recorded interviews. Common problems included crackling or flattening, inaudible voice, background noise, and loss of sound signal. Twelve interviews displayed better sound quality than the other interviews, although our overall results include the measures of vocal parameters for all 28 interviews (as mentioned above, the phone interviews of three participants provided no usable tokens of the /a/ sound).

It was not a goal of this study to compare sound signals recorded by different types of smartphones (refer to Jannetts et al. [18] for such comparison), but rather to compare sound signals (a) captured by audio recorder and smartphone and (b) extracted from different types of audio samples. We were able to select the segments that presented the best sound quality in all interviews, and the measures of the vocal parameters extracted from good sound-quality interviews did not depart significantly from the other interviews. The interviews that produced good sound quality were made with (a) iPhone^®^ smartphones using the TapeACall© application [19] or (b) Android-operated smartphones using the CubeACR© call recorder application [20].

#### Comparisons with Previous Studies

How did our measures of vocal parameters compare with values observed in previous studies? Vocal parameters extracted from the three audio samples had mean values consistent with previous surveys of groups of people who smoked[7–9].^2^ For jitter and shimmer parameters, which measure perturbation in the period and amplitude of the fundamental frequency, these values were higher than those documented in previous studies (e.g. Pinto et al. [7]). With the exception of the fundamental frequency measured in our male participants, and probably owing to the prevalence of participants under the age of 40, overall mean values all depart from values documented for people who do not smoke and/or healthy voices in previous studies [13].

### Fundamental Frequency

Smoking decreases fundamental frequency values. Therefore, we expected to observe lower fundamental frequencies among our participants than in people who do not smoke. This is consistent with results reported in Pinto et al. [7] and Zealouk et al. [8] studies which compared mean fundamental frequencies in groups of adults who smoked and who did not smoke. However, the overall fundamental frequency means that we reported, for all three audio samples, were consistently higher than for the studies [7, 8]. Furthermore, the mean value for the fundamental frequency from our male participants was even higher than the mean value for the group of people who did not smoke in Pinto et al. [7] (114.49 Hz). The overall mean values for the fundamental frequency measured in our study (160.09 Hz for recorded readings and 169.63 Hz for sustained phonation) were also nearer to the overall value of the group of participants who did not smoke in Zealouk et al. [8] (168 Hz), than to that of their group of participants who smoked (143 Hz).

The difference in the mean age of the participants may be the reason for this conflict, since age is also a factor in the value of this vocal parameter, which decreases with age. Two-thirds of our participants were 40 years old or under and had been smoking for less than 20 years. As a result, they had been exposed to fewer smoking years than subjects in previous studies. For example, adults who had been smoking for less than 20 years were excluded from Pinto et al.’s study, whose participants all ranged between 40 and 60 years of age. Young individuals are less likely to suffer from the effect of age on vocal cords and therefore present higher fundamental frequencies than people in old age.

### Formants *F*3 and *F*4

Zealouk et al.’s study [8] returned mean values circa 2500 for *F*3 and 3500 for *F*4, for a group of adults who were smoking at the time of the survey—consisting of 20 men, aged 28–50 with a mean age of 39, and most of them having smoked for at least 13 years. In our study, the overall mean values that we obtained across the three types of recordings were slightly higher for *F*3 and *F*4, but well under the mean values reported by Zealouk et al. for their control group of people who did not smoke: 3000 for *F*3 and 4000 for *F*4. Furthermore, as was mentioned earlier in the discussion, in our study formants *F*3 and *F*4 values were even lower when voice was recorded with a smartphone.

### Jitter and Shimmer

The mean jitter and shimmer values found in our study were high in comparison with the mean values obtained for healthy voices by Teixeira and Fernandes [13]. All three sets of values in our study show some perturbation in the frequency and amplitude of the sound signal.

The overall mean values for the /a/ sound that we logged for jitter and shimmer parameters in our study were higher than the values reported by Pinto et al. [7] for participants who were smoking, with the notable exception of the jitter values that we logged for the sustained phonation of the /a/ sound signal, which were lower than those reported in Pinto et al.’s study.

Jitter and shimmer values were also higher than the values documented by Zealouk et al. [8] for jitter parameters, and on a par with the values they reported for shimmer parameters, although once again the sound phonation departed from this pattern and showed lower values than in Zealouk et al.’s study for jitta, ppq5, shdb, and apq5.

Frequency of the sound signals also appeared to be affected by the use of electronic communication devices and shimmer parameters values were significantly increased for sound signals recorded with smartphones.

### Noise

NHR mean values (0.05–0.23) were equal or higher than the mean of 0.05 (SD: 0.15) documented by Pinto et al. [7] for their group of people who smoked. Our participants’ voices presented noise in the sound signal and a worse voice quality than the group surveyed in [7].

Our participants’ mean HNR values, ranging from 10 to 17.42 across the three types of recordings, were much lower than the 23.9 HNR mean obtained for healthy voices by Teixeira and Fernandes [13], indicating an overall lower efficiency of speech in our participants, across all three types of audio samples.

## Conclusions

Cigarette smoking continues to be the leading preventable cause of death of about 7 million people globally per annum [21]. Many of these deaths occur unnecessarily and prematurely. Earlier diagnosis of smoking-related harm, and subsequent earlier intervention to support abstinence from smoking, could help reduce morbidity and years of life lost due to smoking. The vocal features surveyed in this study have been associated with a clinical outcome, and these biomarkers [22] may be useful for early diagnosis or monitoring of progression of smoking-related diseases.

This study was limited by the small sample size. A further limitation is that a minor number of voice recordings on the smartphone were recorded with an external device rather than a smartphone application. The audio for these may have suffered additional noise and signal distortion. Nevertheless, the results can be used to inform the value of pursuing more costly research and development of diagnostic voice technologies. It is useful to know that the task of self-completing an at-home voice recording was acceptable to a demographically diverse group of adults who smoked. Secondly, the recordings were of sufficient quality for measuring vocal biomarkers using Praat voice analysis software.

Finding that sound signals from spontaneous speech (reading and conversation) can be extracted and usefully analysed is important for advancing the voice diagnosis field. This study contributes to that advancement by demonstrating that audio samples extracted from smartphone-recorded interviews in a naturalistic real-world setting were of sufficient quality to be used for acoustic analysis in Praat. However, our results show that audio signals recorded with a smartphone display significant perturbation, in comparison with signals recorded with an audio recorder. The devices can be calibrated to account for systematic errors, the important thing being that the error remains the same and the random error is not too high.

The results have implications for the advancement of remote delivery of health monitoring and for clinical trials collecting data remotely. The relatively lower cost of collecting vocal biomarker data by smartphone or other digital platforms could be particularly useful for addressing inequities in health diagnosis and cessation support experienced by people living in rural or underserviced areas, such as in low-income countries. Future studies could assess the potential of providing individuals who smoke with feedback on the health of their voice as a tool to enhance risk perceptions and motivate cessation [23]. However, this study suggests that such interventions should focus on people who have smoked heavily and persistently over decades.

This study also provides useful information for the design of future voice diagnostic research. For instance, sustained vowel phonation would provide less intra-participant variability and may be the optimum parameter for analysis of vocal changes among participants in longitudinal studies who overtime may continue to smoke, may switch to vaping or may stop smoking.

## Footnotes

1 However, it is possible to analyse connected speech; this was done with Cepstral peak prominence value in Murton et al. [17].

2 Cepstral peak prominence [17] was not measured in these previous studies. This parameter is also useful at detecting aspects of poor voice quality and could be added in the future studies.
